# Word Embedding for the French Natural Language in Health Care: Comparative Study

**DOI:** 10.2196/12310

**Published:** 2019-07-29

**Authors:** Emeric Dynomant, Romain Lelong, Badisse Dahamna, Clément Massonnaud, Gaétan Kerdelhué, Julien Grosjean, Stéphane Canu, Stefan J Darmoni

**Affiliations:** 1 OmicX Le Petit Quevilly France; 2 Rouen University Hospital Department of Biomedical Informatics, D2IM Rouen France; 3 Rouen University LITIS Laboratory National Institute of Applied Sciences Saint-Étienne-du-Rouvray France; 4 LIMICS Sorbonne Universités Paris France

**Keywords:** natural language processing, data mining, data curation

## Abstract

**Background:**

Word embedding technologies, a set of language modeling and feature learning techniques in natural language processing (NLP), are now used in a wide range of applications. However, no formal evaluation and comparison have been made on the ability of each of the 3 current most famous unsupervised implementations (Word2Vec, GloVe, and FastText) to keep track of the semantic similarities existing between words, when trained on the same dataset.

**Objective:**

The aim of this study was to compare embedding methods trained on a corpus of French health-related documents produced in a professional context. The best method will then help us develop a new semantic annotator.

**Methods:**

Unsupervised embedding models have been trained on 641,279 documents originating from the Rouen University Hospital. These data are not structured and cover a wide range of documents produced in a clinical setting (discharge summary, procedure reports, and prescriptions). In total, 4 rated evaluation tasks were defined (cosine similarity, odd one, analogy-based operations, and human formal evaluation) and applied on each model, as well as embedding visualization.

**Results:**

Word2Vec had the highest score on 3 out of 4 rated tasks (analogy-based operations, odd one similarity, and human validation), particularly regarding the skip-gram architecture.

**Conclusions:**

Although this implementation had the best rate for semantic properties conservation, each model has its own qualities and defects, such as the training time, which is very short for GloVe, or morphological similarity conservation observed with FastText. Models and test sets produced by this study will be the first to be publicly available through a graphical interface to help advance the French biomedical research.

## Introduction

### Context

The use of clinically derived data from electronic health records (EHRs) and other clinical information systems can greatly facilitate clinical research as well as optimize diagnosis-related groups or other initiatives. The main approach for making such data available is to incorporate them from different sources into a joint health data warehouse (HDW), thus containing different kinds of natural language documents, such as prescription, letters, surgery reports—all written in everyday language (spelling errors, acronyms, and short and incomplete sentences).

Clinical named entity recognition (NER) is a critical natural language processing (NLP) task to extract concepts from named entities found in clinical and health documents (including discharge summaries). A semantic health data Warehouse (SHDW) was developed by the Department of Biomedical Informatics of the Rouen University Hospital (RUH), Normandy, France. It is composed of 3 independent layers based on a NoSQL architecture:

- A cross-lingual terminology server, HeTOP, which contains 75 terminologies and ontologies in 32 languages [1]
- A semantic annotator based on NLP bag-of-word methods (ECMT) [2]
- A semantic multilingual search engine [3]

To improve the semantic annotator, it is possible to implement deep learning techniques to the already existent one. To do so, a new text representation, which keeps the most semantic similarities existing between words, has to be designed to fit the input of neural networks algorithms (text embedding).

### Word Embedding

In NLP, finding a text representation that retains the meaning proximities has always been a moot point. Indeed, the chosen representation has to keep the semantic similarities between different words from a corpus of texts to allow indexation methods to output a correct annotation. Thus, the representation of a unique token has to show the proximity with other related meaning concepts (synonyms, hyponyms, cohyponyms, and other related tokens), as illustrated in the quotation “You shall know a word by the company it keeps” [4], now known as the *distributional hypothesis*.

During the 60s, the system for the mechanical analysis and retrieval of text information retrieval system brought the vector space model (VSM), which led to the idea of vectorial representation of words [5,6]. With this approach, the word vectors were sparse (the encoding of a word being a vector of *n* dimensions, *n* representing the vocabulary size). In fact, a compact and precise representation of words could bring several benefits. First comes the computational aspect. Computers are way better to perform operations on low-dimensional objects. This then permits to calculate the probability of a specific concept to appear close to another one. Moreover, the vectors’ dimensions created to represent a word can be used to fit this word in a space and thus make distance comparisons with other tokens. Current unsupervised embedding techniques provide dense and low-dimensional information about a word, either with count-based or predictive-based methods [7]. Different implementations of techniques mapping words into a VSM have been developed.

### Word2Vec

The Word2Vec approach was the first modern embedding released in 2013 [8]. Mikolov et al implemented 2 kinds of architectures: the continuous bag-of-word (CBOW) and the skip-gram (SG).

The *CBOW architecture* is learning to predict a target word *W* by using its context *C*. This model is similar to a feedforward neural network proposed earlier [8,9]. However, the bias brought by the nonlinear layer has been removed with a shared projection layer. The input layer accepts one-hot encoding as input *X_i_* (a sentence is encoded as a very hollow vector. It is composed of 0 or 1, depending on the words found in this sentence and becomes *X’_i_* when passing through the activation function). With a corpus composed of *V* different words and an input layer size of *N* chosen, the hidden representation of this corpus will be a *V × N* matrix with each row representing a word *W_v_* by a vector of dimension *N*. After passing through the linear activation function of the hidden layer, the output *Y_i_* can be computed using the softmax function for each word *W* 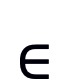
*V*, as described in the equation below [10].

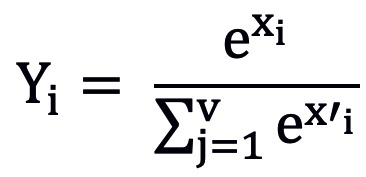



The *SG architecture* uses a given word to predict its context, unlike the CBOW architecture. The entire corpus *V* will thus be transformed into many couples *target* || *context* (*ie, input* || *output* or *x_i_* || *y_i_* of the network) and a stochastic gradient descent optimizing function will be used on this training dataset with a minibatch parsing [11].

Thus, the hidden and the output weight matrix will have a shape of *V × N*, with *N* being again the number of dimensions for word vectors. To reduce the computation of such an amount of data (in a *normal* training, all the weights of the network should be updated for each passage through an example. The amount of changes depends on the size of the contextual windows), the authors brought some new ideas. First, word pairs always appearing together are treated as a single token for both architectures (*New York* is much more meaningful than the combination of *New* and *York*). Then, the frequent words subsampling allows the model to reinitialize a word vector, reducing the over updating of some common words. Finally, the negative subsampling makes the model to update only a portion of the context for each target [12].

### GloVe

This model is the embedding released by Stanford University [13]. Similar to Word2Vec, GloVe can embed words as mathematical vectors. However, it differs on the method used to capture similarity between words, GloVe being a count-based method. The idea was to construct a huge co-occurrence matrix between the words found in the training corpus of shape *V × C* with *V* being the vocabulary of the corpus and *C* being the context examples. The probability *P* (*V_W1_* || *V_W2_*) of a word *V_W1_* being close to another *V_W2_* will increase during the training and fill the co-occurrence matrix. This gigantic matrix is then factorized by using the log function, this idea coming from the latent semantic analysis model [14].

### FastText

It is a newly released model in 2017, which comes from a new idea [15]. Although both Word2Vec and GloVe assumed that a word can be effectively and directly embedded as a vector, Bojanowski et al [15] consider that a word could be the result of all of the vectorial decomposition of this word (subword model). Each word *V_W_* with *V* being the vocabulary can be decomposed into a set of n-characters-grams vectors. For example, the word “boat” can be seen as 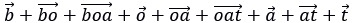
(with the n-gram parameter *n=3*, indicating the maximum number of letters composing a subword). Thus, each word is embedded in the vectorial space as the sum of all vectors composing this token, incorporating morphological information into the representation [16]. Similar to Word2Vec, FastText also comes with the 2 different previously mentioned architectures (SG and CBOW).

### Related Study

For the past few years, the huge interest in word embeddings led to comparison studies. Scheepers et al compared the 3 word embedding methods but these models were trained on different and nonspecific datasets (Word2Vec on news data, whereas FastText and GloVe trained on more academic data, Wikipedia and Common Crawl, respectively, a bias could have been brought by such a difference) [17]. Bairong et al also performed a comparison among these 3 implementations but focused on bilingual automatic translation comparison (BLEU score [18]) and without human evaluation for all the different models. The goal here is to determine the best ability to keep semantic relationships between words [19]. More recently, Beam et al produced huge publicly available word embeddings based on medical data; however, this study did not involve FastText, but involved Word2Vec and GloVe only. Moreover, the benchmark between embedding methods was based on statistical occurrences of the concepts [20]. In a similar way, Huang et al deeply studied Word2Vec on 3 different medical corpuses, measuring the impact of the corpuses’ focus on medicine and without evaluating the semantic relationships [21]. Finally, Wang et al compared word embeddings training set’s influence on models used for different NLP tasks related to medical applications, whereas the goal of this study is to compare embedding implementations trained on the same corpus [22].

Moreover, many different teams or companies have released pretrained word embedding models (eg, Google, Stanford University) that could be used for specific applications. Wang et al also proved that word embeddings trained on a highly specific corpus are not so different than those trained on publicly available and general data, such as Wikipedia [22]. However, in a clinical context, the vocabulary coverage of those embeddings, trained on an academic corpus, is quite low regarding the words used in a professional context. To assess the proportion of these nonoverlapping tokens, 1,250,000 articles’ abstracts were extracted from the French scientific articles database, LiSSa, and they have been compared with the raw health data from the SHDW [23]. These health documents contained 180,362,939 words in total, representing 355,597 unique tokens, and the abstracts from the LiSSa database are composed of 61,119,695 words, representing 380,879 unique tokens. Among the 355,597 unique tokens written in the SHDW documents, 26.11% (92,856/355,597) were not found in the abstracts from the LiSSa corpus (mainly representing misspells, acronyms, or geographic locations). Thus, more than a quarter of the vocabulary used in professional context cannot be better embedded by using an academic pretraining corpus. Thus, local training on specific data is often needed, especially with languages other than English, where less pretrained embedding models are available.

### Contributions

Word embedding comparisons thus have previously been studied, but as far as we know, none of them compared the ability of the 5 actual most used unsupervised embedding implementations trained on a medical dataset produced in a professional context in French, instead of a corpus of academic texts. Moreover, a bias could occur when comparing models trained on different datasets.

Thus, the objective here is to compare 5 different methods (Word2Vec SG and CBOW, GloVe, FastText SG, and CBOW) and to assess which of those models output the most accurate text representation. They will be ranked based on their ability to keep the semantic relationships between the words found in the training corpus. We thus extended the related study by (1) comparing the most recent and used embedding methods on their ability to preserve the semantic similarities between words, (2) removing the bias brought by the utilization of a different corpus to train the compared embedding methods, and (3) using these embedding algorithms on a challenging corpus instead of academic texts.

This representation will then be used as the input of deep learning models constructed to improve the annotating phase, actually performed by the ECMT in the SHDW. This NER phase will be the first step toward a multilingual and multiterminology concept extractor. Moreover, the constructed models will first be available for the community working on medical documents in French through a public interface.

## Methods

### The Corpus

The corpus used in this study is composed of a fraction of health documents stored in the SHDW of the RUH, France. All these documents are in French. They are also quite heterogeneous regarding their type—discharge summaries, surgery or procedure reports, drug prescriptions, and letters from a general practitioner. All these documents are written by medical staff in the RUH and thus contain many typography mistakes, misspells, or abbreviations. These unstructured text files were also cleaned by removing the common header (containing RUH address and phone numbers).

### Documents Deidentification

These documents were then deidentified to protect each identity of every patient or doctor from the RUH. Every first and last name stored in the RUH main databases was replaced by noninformative tokens, such as *<doctor>*, *<firstname>*, or *<lastname>*. Moreover, other tokens have been used, such as *<email>* or *<date>*. In case of a misspelling of a patient’s name in a document or of a lack in the database, a filter based on REGular EXpressions has been defined to catch emails, doctor or professor names (based on the prefix *Dr* or *Prof*, respectively, and their variations), abbreviations such as *Mr* or *Mrs*, dates, and phone numbers without past knowledge. To improve this important phase, a last rule has also been defined. If no patient or doctor name is found in the document, this text is just ruled out to prevent the release of sensitive information in the embedding models.

### Preprocessing

First comes the question about the shape of the input data. Should it be composed of chunks of sentences (data are composed of a list of tokenized sentences) or subsplit by documents (a list of tokenized documents)? The answer to this question depends on what the model will be used for. In our case, the context of each document is important (but not the context of each sentence, which is a good representation for documents dealing with many subjects). Therefore, the input data will be based on document subsplitting.

Then, the data had been lowered (no additional information was brought on word semantics similarity conservation by differences between upper and lower case for this study), the punctuation was removed, and the numerical values were replaced by a meta-token *<number>*. We chose not to remove stopwords because of their negligible impact on the context. Indeed, their multiple apparitions in many different contexts would have just created a cluster of stopwords in the middle of the VSM.

### Training

The models have been implemented thanks to the Gensim Python library [24]. They have been trained on a server powered by 4 XEON E7-8890 v3 and 1To of RAM located on the RUH. We based the tuning of models’ hyperparameters on the literature [25] and on our own experience. The goal here was to compare word embedding implementation; so, we chose to keep equivalent parameters for each model. Chosen values are listed in Table 1.

**Table 1:** Hyperparameters values used to train the 5 word embedding models.

Parameter and applied to model		Value	
**Epochs**		
	Word2Vec/FastText	25
	GloVe	100
**Minimum token count**	
	All 3 models	20
**Context window size**	
	All 3 models	7
**Learning rate**	
	All 3 models	2.5x10^-2^
**Embedding size**	
	All 3 models	80
**Alpha rate**	
	All 3 models	0.05
**Negative sampling**	
	Word2Vec/FastText	12
**Subsampling**	
	GloVe	1e^-6^

### Evaluation

The goal behind these comparisons was to find the model that can represent nonacademic text into a mathematical form, which keeps the contextual information about the words, despite the bias brought by the poor quality of used language. To do so, different metrics have been defined, centered on word similarity tasks. The positive relationships were evaluated with the cosine similarity task and the negative ones with the odd-one task. Analogy-based operations and human evaluation allows us to assess if a given model can keep the deep meaning of a token (antonyms, synonyms, hyponyms, and hypernyms).

### Cosine Similarity

Similarities between the embedded pairs of concepts were evaluated by computing cosine similarity. It has also been used to assess whether the 2 concepts are related or not. Cosine similarity (cos) between word vectors W1 and W2 indicates orthogonal vectors when close to 0 and highly similar vectors when close to 1. It is defined as:

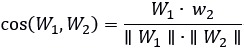



It is possible to define a validation set, composed of couples of terms that should be used in a similar context in our documents (such as *flu* and *virus*). Then, the first token from each couple is sent to each model and the top 10 closest vectors regarding the cosine similarity are extracted. The second word has to be retrieved in these 10 closest vectors to be considered as successful. Then, the total percentage (*p*) of success is calculated regarding the total number of word pairs, with *N* being the number of times the second term had been found in the top 10 closest vectors of the first one with:

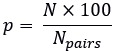



To construct the dataset, 2 well-known validation sets, UMNSRS-Similarity and UMNSRS-Relatedness, were used, containing respectively 566 and 588 manually rated pairs of concepts known to be often found together, [26]. However, our corpus being in French, the translated and aligned version of the MeSH terminology stored in HeTOP was used to translate these 2 sets [27]. The result provided a number of 308 pairs for the UMNSRS-Similarity and 317 pairs for the UMNSRS-Relatedness, the remaining concepts were not directly found in the MeSH.

### Odd One Out Similarity

The odd one out similarity task tries to measure the model’s ability to keep track of the words’ negative semantic similarities by giving 3 different words to the model. Among them, 2 are known as linked, not the third one. Then, the model has to output the word vector that does not clusterize with the 2 others (eg, output car when the input is *car, basketball, tennis*) [28]. To create such a validation corpus, every Medical Sub Heading (MeSH) term appearing more than 1000 times in the corpus has been extracted. The result was a list of 516 MeSH terms, which have been manually clusterized into 53 pairs of linked MeSH concepts according to 2 different medical doctors (MDs). Then, 53 words appearing more than 1000 times in the corpus have been randomly selected to be used as odd terms, one for each pair of MeSH term. The matrix of cosine distance between the 3 tokens was calculated for each item of the odd-one list and for each model. The goal for the model is to output a cosine distance between each of the 2 linked terms and the odd one closer to 0 compared with the one between those 2 linked terms, which should be closer to 1 (indicating more similar vectors). The percentage p of success is then calculated.

### Human Evaluation

A formal evaluation of the 5 methods was performed by a public health resident (CM) and an MD (SJD). A list of 112 terms has been extracted from the MeSH terminology. At least 3 concepts have been extracted from each branch of the MeSH terminology (regardless of branch Publication Characteristics, V). All of these 112 terms have been sent to each model and the top 5 closest vectors regarding the cosine distance have been extracted from every model. Overlapping top-close vectors between models were grouped, avoiding to evaluate several times the same answer and the total list was randomized to avoid the annotator’s tiredness. CM and SJD then blindly assessed the relevance of each vector compared with the sent token. These citations were assessed for relevance according to a 3-modality scale used in other standard Information Retrieval test sets: bad (0), partial (1), or full relevance (2).

### Analogy-Based Operations

Mikolov’s paper presenting Word2Vec showed that mathematical operations on vectors such as additions or subtractions are possible, such as the famous (*king–man*)+*woman~queen*. This kind of task helps check the semantic analogy between terms. With Mikolov’s operation, it is possible to affirm that *king* and *man* share the same relationship properties as *queen* and *woman*. To check the conservation of these properties by each model, several mathematical operations covering a wide range of possible subjects found in the EHR (hospital departments, human tissues, biology, and drugs) were defined following Mikolov’s style ([*Term 1 – Term 2*] + *Term 3 ~ Term 4)*). Then, the operation was performed using vectors *Term 1*, *Term 2*, and *Term 3* extracted from each model. The resulting vector was compared with the *Term 4* vector, the operation being considered as correct if this *Term 4* vector was found to be the closest one regarding the cosine distance with the operation resulting one, indicating a semantic similarity between *Term 3* and *Term 4*, similar to the one between *Term 1* and *Term 2*.

### Word Clusters

In the VSM, words are grouped by semantic similarity, but the context does influence this arrangement a lot. Every model’s vector dimensions have been reduced and projected on 2 dimensions using the t-SNE algorithm. Then, logical word clusters have been manually searched in the projection. This step was not a part of the global final score but allowed for the rapid assessment of the quality of a word representation.

### Going Further: Model Improvement

To check if a model pretraining affected the result or not, a new version of the best model regarding the tasks explained above was trained twice. First, the French paper abstracts from the LiSSa corpus (1,250,000 in total) were used for model pretraining. Then, this resulting embedding was trained a second time on the documents from the RUH without changing any parameter. All of the automatic tests were performed for this model a second time to assess if the added academic data improved the model’s quality regarding our evaluation.

## Results

### The Corpus

In total, 641,279 documents from the RUH have been de-identified and preprocessed. With regard to the vocabulary, texts have been split into 180,362,939 words in total, representing 355,597 unique tokens. However, this number can be pondered with 170,433 words appearing only once in the entire corpus (mainly misspells, but also geographic locations or biological entities, such as genes and proteins). In total, 50,066 distinct words were found more than 20 times in the corpus, thus present in the models (minimum count parameter set to 20). On average, each document contains 281.26 words (*SD* 207.42). The 10 most common words are listed in Table 2.

**Table 2:** The 10 most common words of our corpus. Note that Rouen is the city where the training data come from.

French	English	Occurrences
de	of	9,501,137
docteur	doctor	4,822,797
le	the	3,975,735
téléphone	phone	3,147,286
d’	’s	3,036,198
Rouen	Rouen	2,763,918
à	at	2,271,317
l’	the	2,129,090
et	and	2,091,502
dans	in	2,001,135

**Figure 1:**
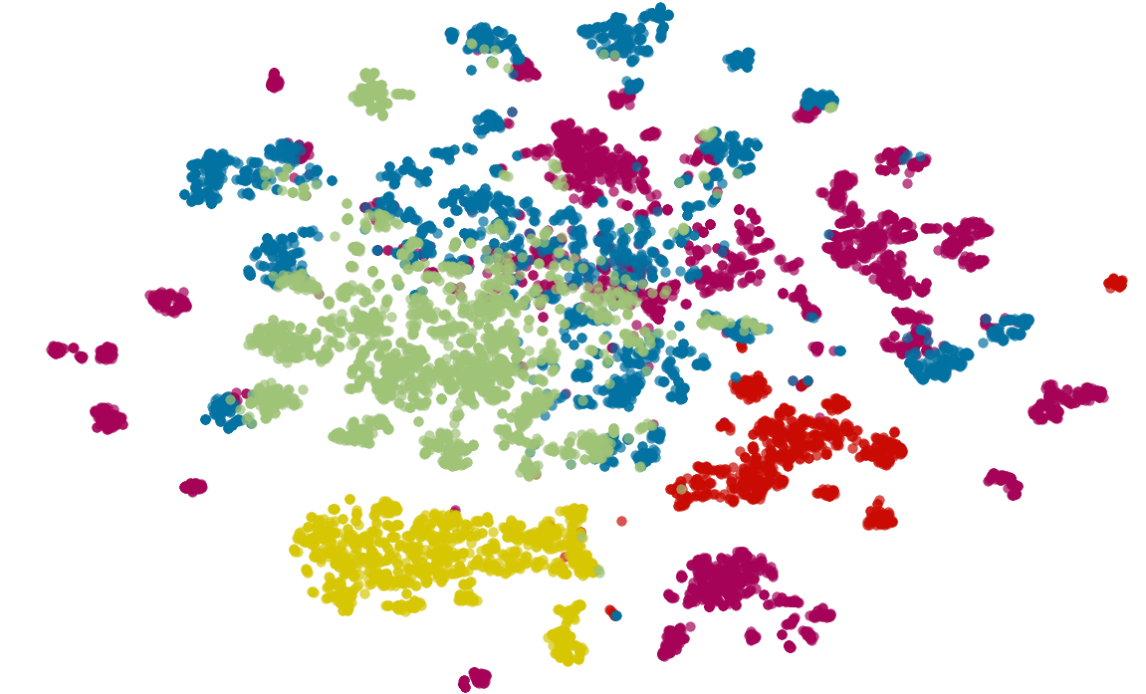
Two-dimensional t-SNE projection of 10,000 documents randomly selected among main classes in the HDW. The five different colors correspond to the five types of documents selected (discharge summaries [green], surgery [blue] or procedure [purple] reports, drug prescriptions [yellow], letters from a general practitioner [red]).

These documents were decomposed using the Term-Frequency Inverse-Document-Frequency (TF-IDF) algorithm that resulted in a frequency matrix. Each row, representing an article, had been used to cluster those documents with a kMeans algorithm (number of classes *K*=5). To visualize their distribution on 2 dimensions, t-SNE algorithm had been used (Figure 1) [29].

Those main classes were well separated, thus the vocabulary itself contained in the documents from the HDW was sufficient to clusterize each type of text. However, discharge summaries, surgery, or procedure reports were a bit more mixed because of the words used in these kinds of context (short sentences, acronyms and abbreviations, and highly technical vocabulary). With regard to drug prescriptions and letters to a colleague or from a general practitioner, they present a more specific vocabulary (drugs and chemicals and current/formal language, respectively), involving more defined clusters for these 2 groups.

### Training

Regarding the training time, models were very different. GloVe was the fastest algorithm to train with 18 min to process the entire corpus. The second position was occupied by Word2Vec with 34 min and 3 hours 02 min (CBOW and SG architectures, respectively). Finally, FastText was the slowest algorithm with a training time of 25 hours 58 min with SG and 26 hours 17 min with CBOW (Table 3).

**Table 3:** Algorithms training time (min).

Algorithm	Training time (min)
FastText SG	1678.1
FastText CBOW	1577.0
Word2Vec SG	182.0
Word2Vec CBOW	33.4
GloVe	17.5

**Table 4:** Percentage of pairs validated by the 5 trained models on 2 UMNSRS evaluation sets.

Algorithm	UMNSRS-Sim	UMNSRS-Rel
FastText SG	3.89	5.04
FastText CBOW	3.89	3.79
Word2Vec CBOW	3.57	4.10
Word2Vec SG	2.92	4.10
GloVe	1.29	0.94

**Table 5:** Percentage of odd one tasks performed by each of the 5 trained models.

Algorithm	Odd one
Word2Vec SG	65.4
Word2Vec CBOW	63.5
FastText SG	44.4
FastText CBOW	40.7
GloVe	18.5

GloVe performs much better in terms of computational time because of the way it handles the vocabulary. It is stored as a huge co-occurrence matrix and thanks to its count-based method, which is not computationally heavy, it can be highly parallelized. It was expected that FastText would take a lot of time to train, because of the high number of word subvectors it creates. However, for Word2Vec, the difference between the 2 available subarchitectures is highly visible (33 min to 3 hours 02 min). This difference could come from the hierarchical softmax and one-hot vector used by the CBOW architecture, which reduces the usage of the CPU. With SG, the minibatch parsing of all the *context* || *target* pairs highly increases the time to go through all possibilities.

### Evaluation

### Cosine Similarity

The total number of UMNSRS pairs successively retrieved by each model has been extracted (308+317 pairs in total with UMNSRS-Rel and UMNSRS-Sim). The percentages of validated pairs from the UMNSRS datasets are presented in the Table 4. FastText SG performed this task with the highest score (3.89% and 5.04% for UMNSRS-Sim and UMNSRS-Rel, respectively). The very low scores indicate that this kind of published dataset is useful to validate models trained on more academic texts.

### Odd One Similarity

With regard to the odd one similarity task, models are quite different (Table 5). Word2Vec is the best so far with 65.4% and 63.5% of odd one terms correctly isolated with SG and CBOW architectures, respectively. Both the FastText architectures achieved a score between 44.4% (SG) and 40.7% (CBOW). GloVe only found the correct odd terms in 18.5% of the tested tasks.

With regard to the subarchitectures presented by both Word2Vec and FastText, the SG always performed better than the CBOW, possibly because of the negative sampling. Indeed, the studied corpus is quite heterogeneous and words can be listed as items (eg, drugs) instead of being used in correct sentences. Thus sometimes, the complete update of vectors’ dimensions generates non-senses in the models (items from lists are seen as adjacent by the models, thus used in same sentences, leading to non-senses).

### Human Validation

**Figure 2:**
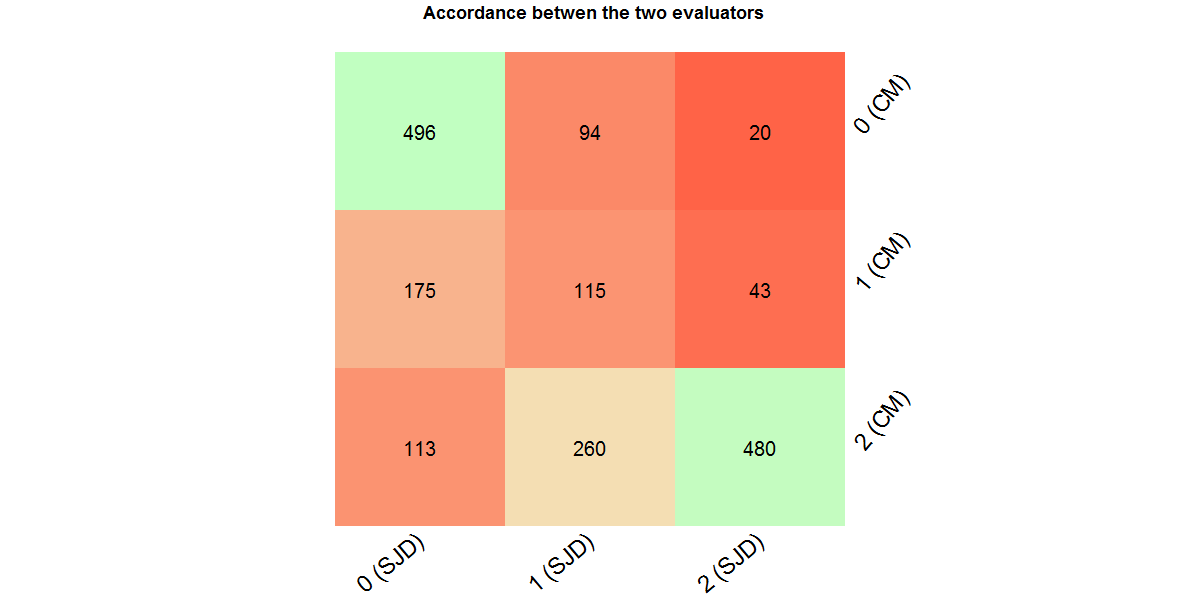
Global representation of the notation agreement between the 2 evaluators (CM and SJD). Notes attributed to a model output are going from 0 (bad matching) to 2 (good matching). Colors are ranging from light green (high agreement) to red (low agreement).

The evaluation focused on 1796 terms (5 vectors, 112 MeSH concepts, 5 models, and 1004 terms were returned multiple times by different models) rated from 0 to 2 by 2 evaluators. First, the agreement between CM and SJD was assessed with a weighted kappa test [30]. A kappa (k)=.6133 was obtained. According to the literature, the agreement between the 2 evaluators can be considered as substantial [31]. This agreement can be retrieved in Figure 2. The accord is stronger for the extreme scores (0 and 2) whereas the agreement about the middle score of 1 is least pronounced.

Moreover, to assess if human evaluators remained coherent regarding the cosine distance computed by each model, the average note given by the 2 evaluators was compared with the average of the cosine distance computed for each model (Table 6). Word2Vec with the SG architecture performed the highest score, regardless of the evaluator (1.469 and 1.200). Interestingly, GloVe computed the closest to 1 cosine distance in averages (0.884 on the top 5 terms to each of the 112 given concepts, indicating the highest similarity), whereas both evaluators gave it the lowest grade.

**Figure 3:**
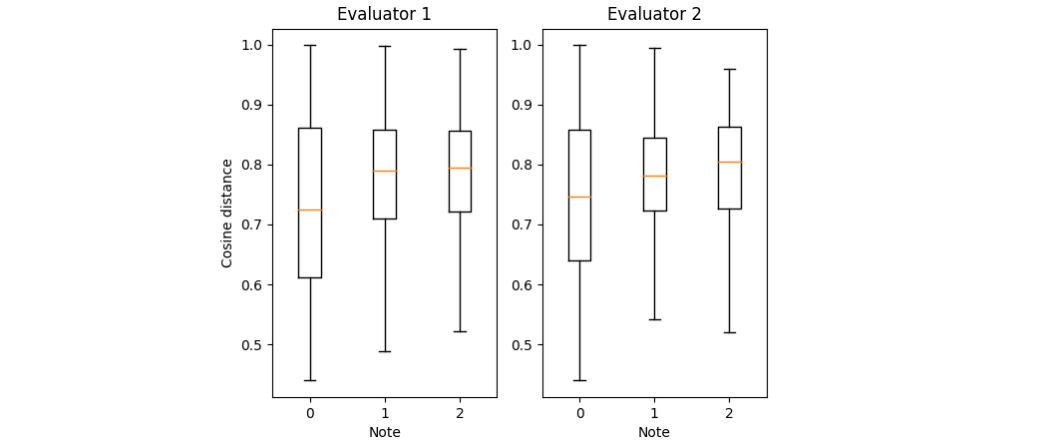
Comparison of the cosine distance calculated regarding the note given by two human evaluators. In both cases, the lower the note is, the lower the average distance is (evaluator 1, SJD; evaluator 2, CM).

To go further, the cosine distances between the 112 sent concepts and the 1796 returned were plotted for each of the 3 modalities rated by the evaluators (Figure 3). In fact, when humans are judging the quality of a returned vector as poor (note 0), the cosine distance between this vector and the queried one is also lower and vice-versa.

**Table 6:** Comparison between cosine distance computed by each model and the human evaluation performed (notes ranging from 0 to 2). Notes and distances are in averages on the top 5 closest vectors for 112 queries on every model by each of the 2 evaluators (evaluator 1, SJD; evaluator 2, CM).

Model	Cosine	Evaluator 1	Evaluator 2
Word2Vec SG	0.776	1.469	1.200
Word2Vec CBOW	0.731	1.355	1.148
FastText SG	0.728	1.200	1.111
FastText CBOW	0.748	1.214	1.048
GloVe	0.884	0.925	0.480

### Analogy-Based Operations

A list of 6 mathematical operations has been defined with the help of an MD and a university pharmacist (listed in Textbox 1). Each operation consists in verifying if 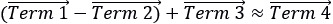, allowing to check if the similarity between *Term 1* and *Term 2* is the same as the one between *Term 3* and *Term 4*. These operations have been defined to cover a wide range of subjects (RUH departments, drugs, and biology).

Each operation 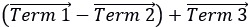
has been performed on vectors from each model and the nearest vector to the resulting one has been extracted. Regarding this task, Word2Vec got the highest score on this task (especially for SG architecture (5/6), while CBOW only reached (3/6)). FastText, independently of the architecture studied, obtained a score of (3/6). GloVe got the lowest score by reaching (2/6).

Interestingly, no operation has been failed by the 5 models, indicating that none of them is simply not logical or just too hard to perform for word embedding models. Operation 2 has been missed by both Word2Vec and FastText SG, whereas CBOW architectures succeeded to perform it for both algorithms. In the corpus, tumors (*mélanome* [*melanoma*] and *adénome* [*adenoma*]) were cited far from their localization (*peau* [*skin*] and *glande* [*gland*], respectively). This distance may be too high for the context-window size (7 words).

GloVe only performed operations 1 and 5. Only Word2Vec SG succeeded on the 5th one. The low score for this task can come from the fact that GloVe treated only pairs of words in the co-occurrence matrix. Thus, relations in common between 2 tokens and a third one are not taken in account.

FastText algorithm just got the average score with SG and CBOW. They both failed to perform operations number 4 and 5 (also number 2 for SG and number 3 for CBOW). The subword decomposition performed by this algorithm was keeping track of the context, but was not as accurate as Word2Vec SG in this task. This imbalance was not compensated by the SG architecture, which performed better for Word2Vec, indicating that this subword decomposition has a really strong impact on the embedding.

**Relation number and mathematical operation performed. For each relation number, the first line is in French and the second line is in English.**

1. (cardiologie - coeur) + poumon ~ pneumologie
  (cardiology - heart) + lung ~ pneumology
2. (mélanome - peau) + glande ~ adénome
  (melanoma - skin) + gland ~ adenoma
3. (globule - sang) + immunitaire ~ immunoglobuline
  (corpuscle - blood) + immune ~ immunoglobulin
4. (rosémide - rein) + coeur ~ fosinopril
  (furosemide - kidney) + heart ~ fosinopril
5. (membre - inférieur) + supérieur ~ bras
  (limb - lower) + upper ~ arm
6. (morphine - opioide) + antalgique ~ perfalgan
  (morphine - opioid) + antalgic ~ perfalgan

### Word Clusters

**Figure 4:**
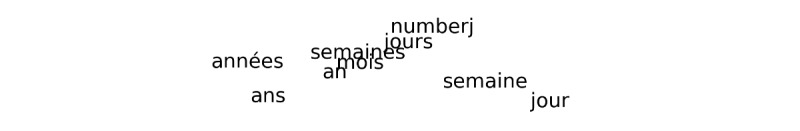
Small cluster of words found in both Word2Vec SG and CBOW (second one shown). Année(s) and an(s) mean year(s), semaine(s) mean week(s) and jour(s) mean day(s). The meta-token "number" used to replace numbers is visible in the expression numberj.

As a visual validation, t-SNE algorithm was applied on vectors extracted from each of the 5 models. To investigate how word vectors are arranged, clusters had been manually searched on the projection. Word2Vec clustered words with a good quality regarding the context they could be used in. Both SG and CBOW architectures had logical word clusters, for example, related to time (Figure 4).

Many other clusters were found by reducing the dimension of both Word2Vec SG and CBOW results; some are showed on Multimedia Appendix 1. These clusters of linked words were underlying the fact that the context in which words are used has a strong impact on the words’ vectorization for this algorithm. In Figure 4, it is easily visible that the word structure itself (word size and the letters composing it) does not influence the representation of words produced by Word2Vec at all. In fact, tokens seen in this insert are very different, regarding the size (ranging from 2 letters for *an* [*year*] to 8 for *semaines* [*weeks*]) or the composition of letters (no letters in common between the 2 neighbors *semaine* [*week*] and *jour* [*day*]).

**Figure 5:**
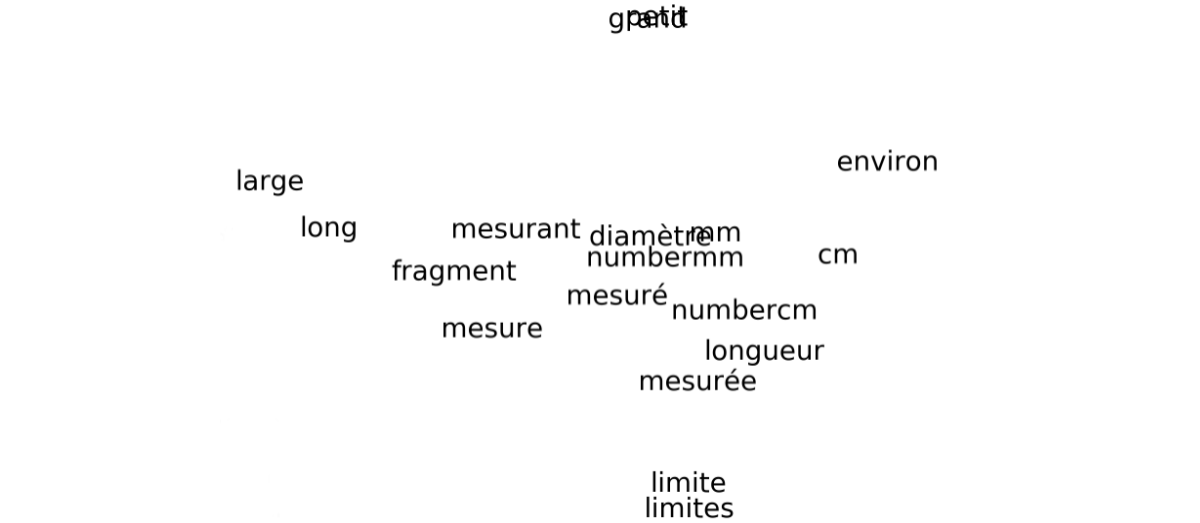
Cluster of words related to the size found by reducing the number of dimensions of word vectors produced by GloVe algorithm.

By looking at the dimensional reduction of vectors produced by GloVe, it is visible how co-occurence matrix used by this algorithm is affecting the placement of vectors in the VSM. In fact, words often used close to each other (and not especially on the same context, such as Word2Vec) are clusterizing well. In the group given as an example in Figure 5, it is visible that sentence segments are almost found intact. Indeed, the large co-occurrence matrix very well captures the similarities found inside the sliding window, but 2 words having the same meaning but not found in the same context (ie, surrounded by other different tokens) will have more difficulties to clusterize with this algorithm.

With regard to FastText, it is interesting to notice that clusters of words used in a similar context were found but other variables do influence the spatial arrangement of the vectors a lot when projected on 2 dimensions: word size and composition. Indeed, as seen on the Multimedia Appendix 2, a gradient starting from the edges of the word projection to the center is following the size of tokens. The shortest ones are found on the edges whereas the longest, in the middle, indicating that the subword vectors created by FastText to decompose each word are strongly impacted by the morphological structure of embedded words.

With regard to the global shape of the 5 projections on the Multimedia Appendix 3, no meaningful distinction can be made between the 5 studied models at this scale. The diversity found at a local scale is not projected on the global one.

**Figure 6:**
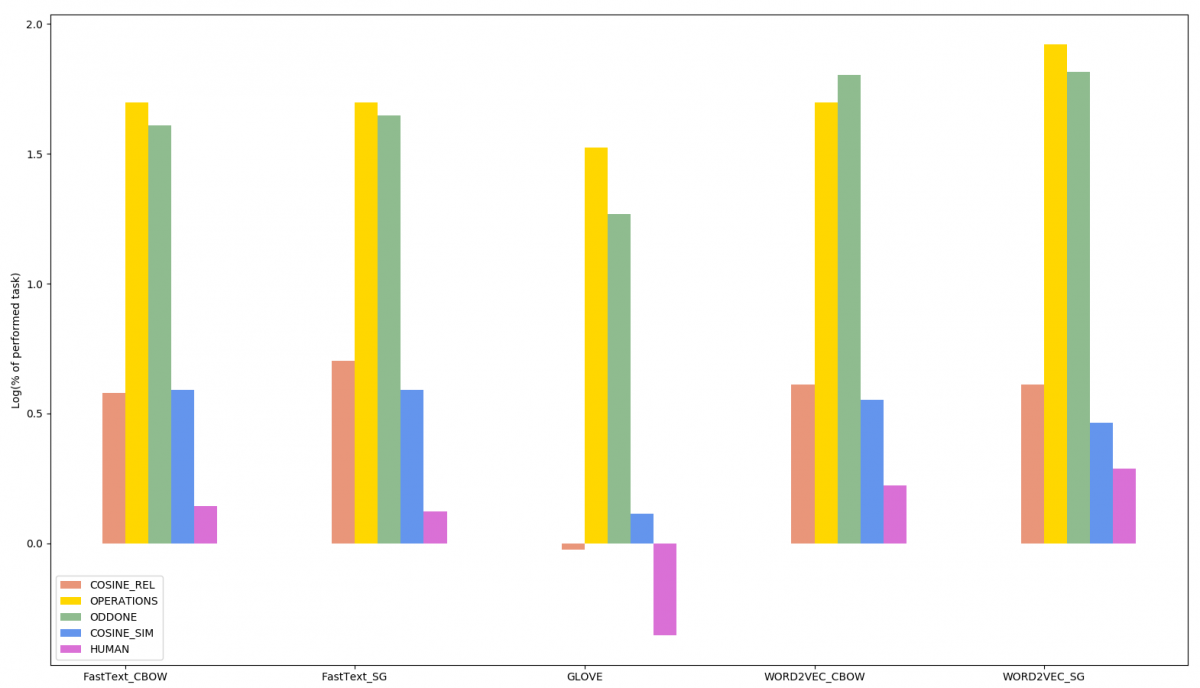
Pulled scores for each task regarding every of the five trained models. Log has been used to facilitate the visualization. Cosine score is duplicated regarding the UMSNRS used set.

### Model Improvement

So far, Word2Vec with the SG architecture showed the best results in average (Figure 6). Thus, a subset of 350,000 French abstracts has been extracted from the LiSSa database, hosted at the RUH, to pretrain this embedding model. It took nearly 20 min for the algorithm to preprocess these data with the same workflow than the one presented in the method section and to train on it (parameters listed in Table 1). Afterward, another 48 min were needed to update word vectors, thanks to the 607,135 health documents contained in the HDW from the RUH.

When this model trained on 2 different datasets is compared with the initial Word2Vec model (without any pretraining), scores were not changed with regard to the cosine and odd one tests (4.1% on the UMNSRS-Rel and 65.4%, respectively). Interestingly, the grade coming from analogy-based operations decreased, lowered from 5/6 to 3/6. This could come from the fact that documents used for pretraining (scientific articles) were highly specialized in a domain, leading to already strongly associated vectors.

## Discussion

### Principal Findings

In this study, the 3 most famous word embeddings have been compared on a corpus of challenging documents (2 architectures, each for Word2Vec and FastText, as well as GloVe) with 5 different evaluating tasks. The positive and negative semantic relationships have been assessed, as well as the word sense conservation by human and analogy-based evaluation.

The training in our 600,000 of challenging documents showed that Word2Vec SG got the best score for 3 on the 4 rated tasks (FastText SG is the best regarding the cosine one). These results are coherent with those obtained by Th et al, who compared Word2Vec and GloVe with the cosine similarity task [32]. More specifically, the CBOW architecture is training way faster, whereas the SG is more accurate on semantic relationships. This model seems to be more influenced by the context in which each word is used, than by the word composition itself. GloVe got the worst grade regarding to our evaluations; however, it is the fastest to train so far. Moreover, GloVe was the only one not implemented in the Python library *Gensim*, which could have brought a bias in this study. This model is computing a cosine distance closer to 1 in average between queried word and close ones, whereas human judgment shows the lowest grade. With regard to FastText, it is interesting to notice that the morphological similarities are kept in account in the vector space creation. In fact, word clusters are highly impacted by the word’s composition in letters and by its size. However, the subvector decomposition of words allows this kind of model to be queried by words absent in the original training corpus, which is impossible with others. Therefore, this model could be used for orthographic correction or acronym disambiguation, for example.

The medical corpus used as a training set for these embedding models is coming from a real work environment. First, finding a good evaluation for embeddings produced in such a context is a hard task. The performances shown by some models trained on scientific literature or on other well-written corpus should be biased regarding their utilization on a very specific work environment. Second, based on our results, an amount of 26.1% of unique tokens found in the health-related documents are not present in an academic corpus of scientific articles, indicating a weakness of the pretrained embedding models. Documents produced in a professional context are highly different compared with this kind of well-written texts. Finally, in this study, pretraining an embedding with an academic corpus and then on the specific one does not improve the model’s performances. It even lowers the score associated to analogy-based operations, indicating strongly associated vectors in the VSM, which leads to a loose of the inherent plasticity of this kind of model to deeply understand the context of a word.

### Limitations

There are a few limitations in our study. First, other embedding models, newly released, could have been compared as well (BERT [33] and ELMo [34]). Second, other clinical notes from different health establishments could have been joined to this study, to investigate how the source of the corpus could affect the resulting similarities found in the embedding space. The complete comparison could also have been trained on nonclinical data, which are highly sensitive and hard to obtain, to help reproducibility. Finally, the quality of those embedding has been checked regarding the semantic similarity conservation, but other metrics could affect this judgment, depending on the model’s usage.

Regarding the cosine annotation, low scores could be explained by the number of occurrences of each term from the 625 words pairs in the corpus of texts. The UMNSRS-Rel dataset contains 257 unique terms for 317 word pairs, whereas the UMNSRS-Sim contains 243 terms for 308 word pairs. First, 128 words in total (25.6%) have been found less than 20 times regarding all of the 641,279 documents, thus being absent in the model because of the *min_count* parameter. These words are found in 452 word pairs in total (231/317 in the UMNSRS-Rel and 221/308 in the UMNSRS-Sim), representing 72.3% of the total number of word pairs searched that cannot be found in the models.

Most of the words absent from the models are drugs’ molecular names, whereas practitioners from the RUH often use the trade names to refer to a drug (eg, ESPERAL instead of *disulfirame*). The natural medical language used in the RUH by the practitioners prevents some words to be found: use of an acronym (HTA instead of *hypertension artérielle*, meaning *hypertension*) or of a synonym (*angor* instead of *angine de poitrine*, meaning *angina pectoris*). Another explanation could come from the fact that some associations defined in those UMNSRS datasets can be true in an academic context, but will be very rarely found in a professional context.

With a median number of occurrences of 230 in the entire corpus of health documents, 176 words (28.1%) have been found more than 1000 times. Whereas the biggest proportion of the low-frequency words was composed of drugs or molecules names, the high-frequency group of words (up to 134,371 times for the word *douleur*, meaning *pain*) is mainly composed of clinical symptoms or diseases. This validation corpus seems to be just not suitable to investigate the quality of embedding trained on such a corpus.

## Conclusions

In our case, Word2Vec with the SG architecture got the best grade in 3 out of the 4 rated tasks. This kind of embedding seems to preserve the semantic relationships existing among words and will soon be used as the embedding layer for a deep learning based semantic annotator. More specifically, this model will be deployed for semantic expansion of the labels from medical controlled vocabularies. To keep the multilingual properties of the actual annotator, a method of alignment between the produced embedding and other languages will also be developed. Other recently tested unsupervised embedding methods exhibit a certain quality, but their ability to preserve the semantic similarities between words seems weaker or influenced by other variables than word context.

As soon as the paper is submitted, any end user will be able to query the word embedding models produced by each method on a dedicated website as well as to download high quality dimension reduction images and test sets [35]. This embedding will be the first publicly published embedding in French, allowing the NLP medical research on French language to go further.

## Appendix

Multimedia Appendix 1 Other word clusters found in the Word2Vec model (CBOW architecture). Red words represent departments from the RUH (cardiology, gynecology, pneumology, etc.) while the red circle indicate months of year. These two groups are near because of the appointment letters or the summary of patients' medical background found in the corpus. Only words appearing more than 5,000 times in the entire corpus have been plotted.

Multimedia Appendix 2 Words size gradient visible while projecting FastText model in two dimensions. In the background is the entire model, in the front the middle-right squared piece zoomed. Red words correspond to units for International Systems. They are grouped with two or three-letters words, while words visible on the left are longer. Only tokens appearing more than 5,000 times in the entire corpus have been plotted.

Multimedia Appendix 3 Global shape of the cloud generated by the dimension reduction by t-SNE of the five VSM created by the five trained word embedding models. Clouds design is highly similar; however, Word2Vec CBOW (figure B) seems to be more compact regarding the y axis compared to the other four. A: Word2Vec SG; B: Word2Vec CBOW; C: GloVe; D: FastText SG; E: FastText CBOW.

## Abbreviations

CBOW: continuous bag-of-word
EHR: electronic health records
HDW: health data warehouse
MD: medical doctor
NER: named entity recognition
NLP: natural language processing
RUH: Rouen university hospital
SG: skip-gram
SHDW: semantic health data warehouse
VSM: vector space model
