# Supplementary figures and images for: Word Embedding for the French Natural Language in Health Care: Comparative Study
Source: JMIR Med Inform. 2019 Jul 29;7(3):e12310. doi: 10.2196/12310 (PMC6690161; doi:10.2196/12310)

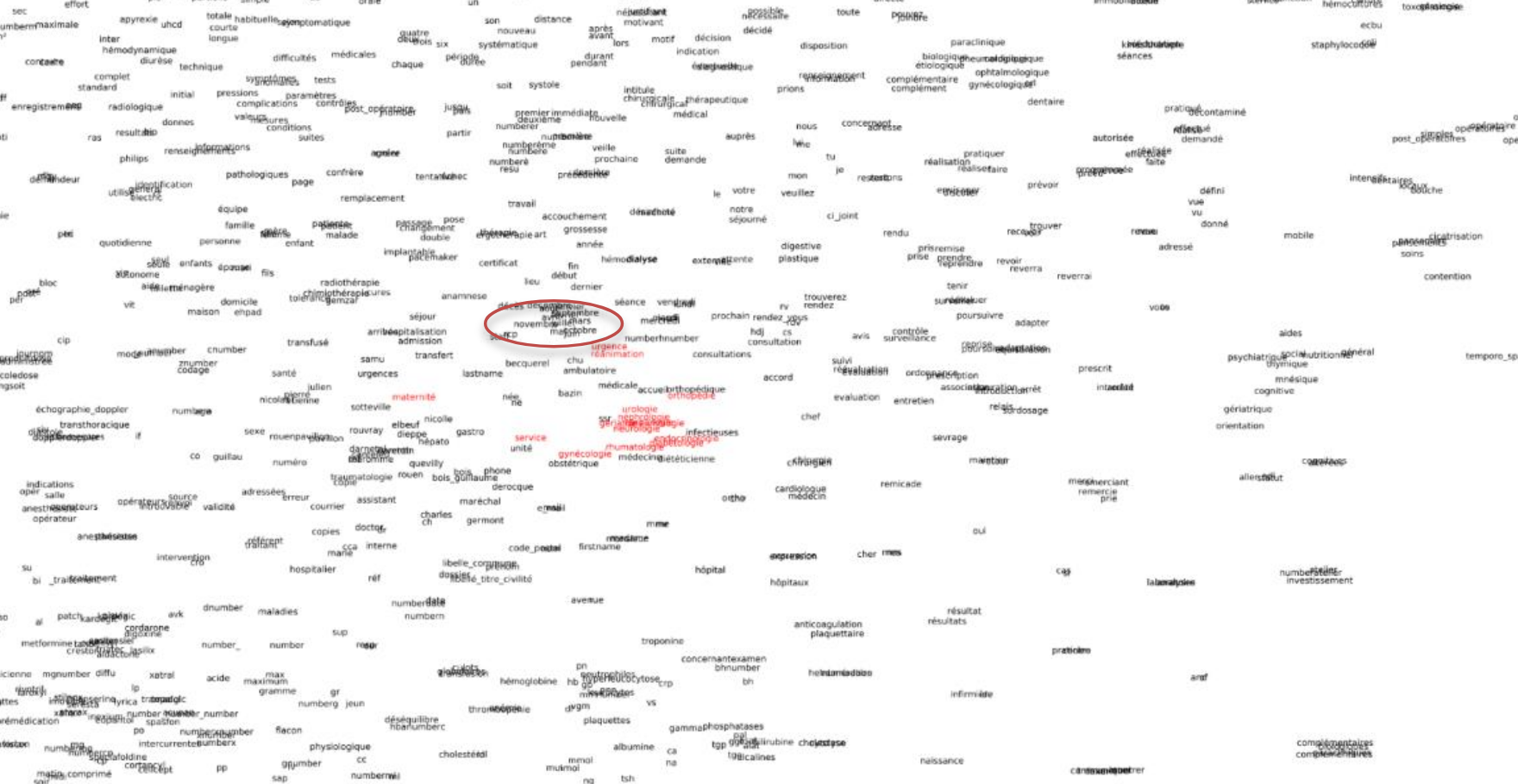

Supplement: Multimedia Appendix 1 [file medinform_v7i3e12310_app1.png]

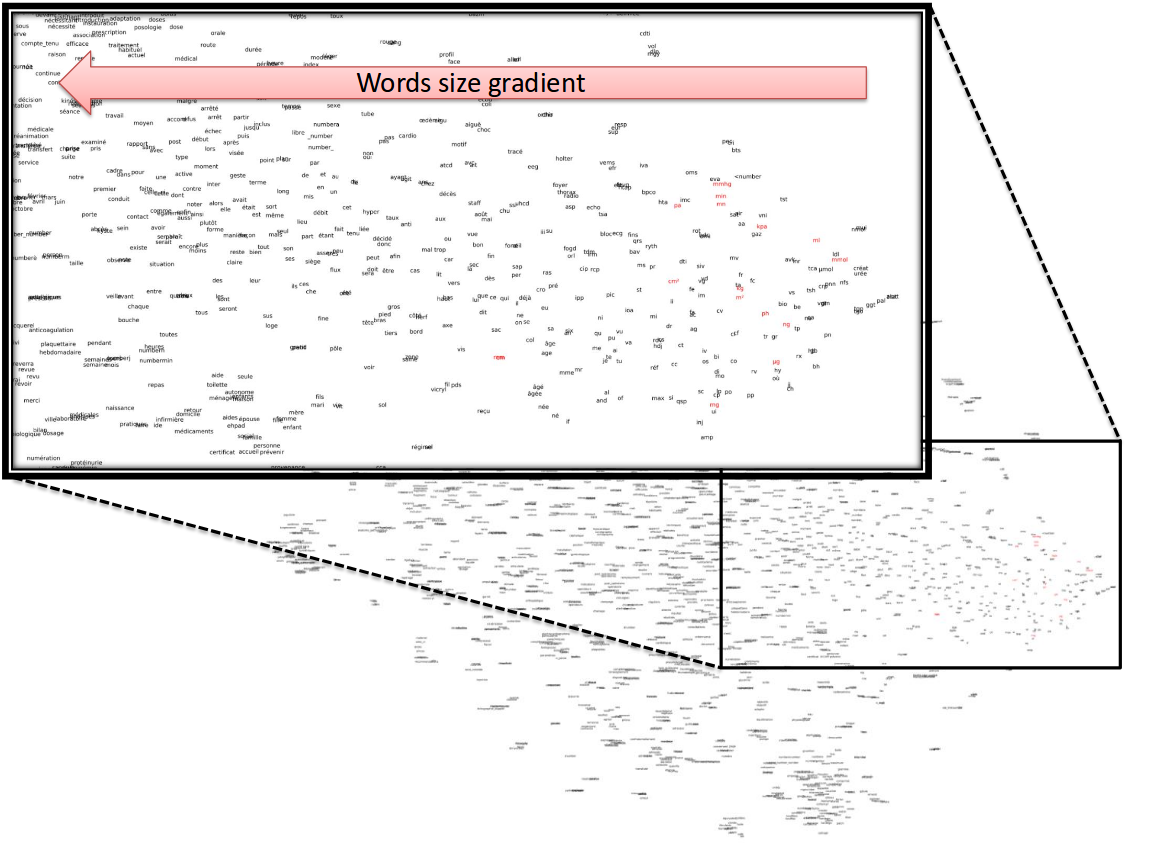

Supplement: Multimedia Appendix 2 [file medinform_v7i3e12310_app2.png]

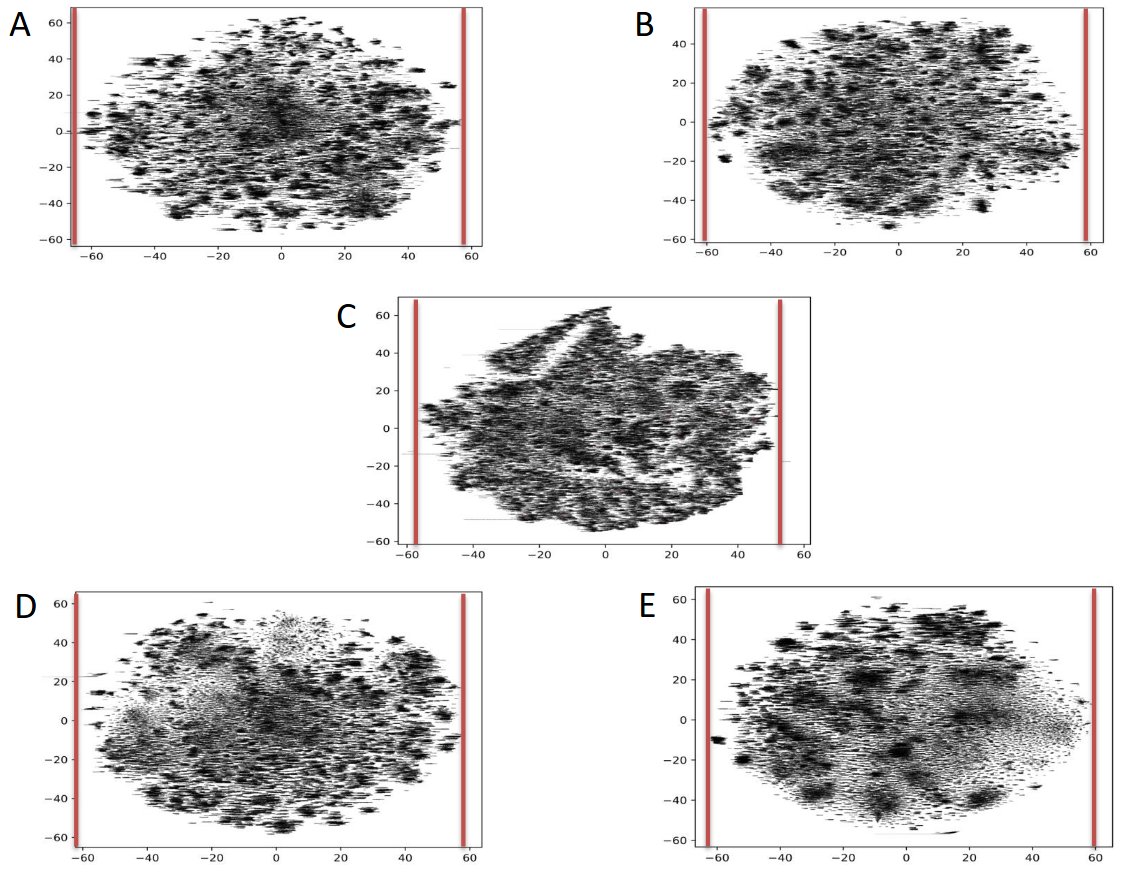

Supplement: Multimedia Appendix 3 [file medinform_v7i3e12310_app3.png]
